# Comparative *In Vivo* Biocompatibility of Cellulose-Derived and Synthetic Meshes in Subcutaneous Transplantation Models

**DOI:** 10.1021/acs.biomac.4c00984

**Published:** 2024-10-08

**Authors:** Nina M. M. Peltokallio, Rubina Ajdary, Guillermo Reyes, Esko Kankuri, Jouni J. T. Junnila, Satu Kuure, Anna S. Meller, Jani Kuula, Eija Raussi-Lehto, Hannu Sariola, Outi M. Laitinen-Vapaavuori, Orlando J. Rojas

**Affiliations:** † Department of Equine and Small Animal Medicine, Faculty of Veterinary Medicine, University of Helsinki, Viikintie 49, FI-00014 Helsinki University, Finland; ‡ Biobased Colloids and Materials, Department of Bioproducts and Biosystems, School of Chemical Engineering, Aalto University, P.O. Box 16300, FI-00076 Aalto, Espoo,Finland; § Department of Pharmacology, Faculty of Medicine, University of Helsinki, P.O. Box 29, Helsinki 00014, Finland; ∥ EstiMates Oy, Kamreerintie 8, FI-022770 Espoo, Finland; ⊥ GM unit, Helsinki Institute of Life Science/STEMM, Research Program′s Unit, Faculty of Medicine, University of Helsinki, P.O. Box 63, Helsinki 00014, Finland; # Laboratory Animal Centre, HiLIFE, University of Helsinki, P.O. Box 29, Helsinki 00014, Finland; ∇ Department of Neuroscience and Biomedical Engineering, School of Science, Aalto University, P.O. Box 16300, FI-00076 Aalto, Espoo, Finland; ○ Customer-oriented Wellbeing and Health Services, Metropolia University of Applied Sciences, PL 4000, FI-00079 Metropolia, Helsinki,Finland; ◆ Department of Pathology, Faculty of Medicine, University of Helsinki, P.O. Box 63, Helsinki 00014, Finland; ¶ Bioproducts Institute, Department of Chemical and Biological Engineering, The University of British Columbia, 2360 East Mall, Vancouver, BC V6T 1Z3, Canada; †† Department of Wood Science, University of British Columbia, 2385 East Mall, Vancouver, BC V6T 1Z4, Canada; ‡‡ Department of Chemistry, University of British Columbia, 2036 Main Mall, Vancouver, BC V6T 1Z1, Canada

## Abstract

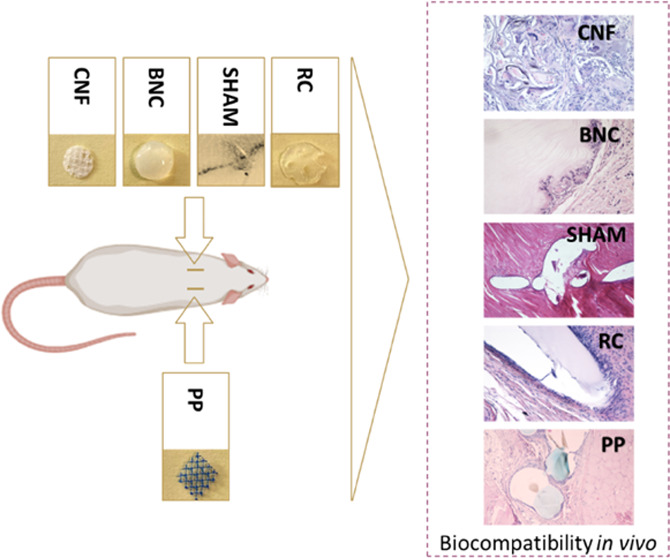

Despite the increasing interest in cellulose-derived materials in biomedical research, there remains a significant gap in comprehensive *in vivo* analyses of cellulosic materials obtained from various sources and processing methods. To explore durable alternatives to synthetic medical meshes, we evaluated the *in vivo* biocompatibility of bacterial nanocellulose, regenerated cellulose, and cellulose nanofibrils in a subcutaneous transplantation model, alongside incumbent polypropylene and polydioxanone. Notably, this study demonstrates the *in vivo* biocompatibility of regenerated cellulose obtained through alkali dissolution and subsequent regeneration. All cellulose-derived implants triggered the expected foreign body response in the host tissue, characterized predominantly by macrophages and foreign body giant cells. Porous materials promoted cell ingrowth and biointegration. Our results highlight the potential of bacterial nanocellulose and regenerated cellulose as safe alternatives to commercial polypropylene meshes. However, the *in vivo* fragmentation observed for cellulose nanofibril meshes suggests the need for measures to optimize their processing and preparation.

## Introduction

The biomedical field is on a quest for sustainable and biocompatible materials that integrate seamlessly with the human body.^1,2^ With the increasing prominence of such materials, extensive research has focused on understanding the complex interplay between implanted biomaterials and host tissue.^3,4^ The ideal postimplantation scenario anticipates these materials to induce a transient inflammatory response,^4^ which aids the body’s long-term healing mechanisms without causing undesirable short- or long-term complications.^5^

Historically, synthetic meshes, particularly those composed of polypropylene (PP), have led surgical treatments, such as hernia and pelvic organ prolapse repairs, since the 1950s.^6−8^ However, the widespread use of PP meshes is now being challenged or halted due to inconsistencies in outcomes,^6,7,9^ including significant patient-reported complications and adverse reactions.^7,10−12^ Regulatory interventions, such as the market removal of certain PP mesh products,^13^ highlight these concerns. Nevertheless, the search for the ideal mesh—combining biocompatibility, mechanical durability, and resistance—continues.^5,7,14,15^

Cellulose is frequently noted for its economic viability and inherent nontoxicity.^1,16,17^ As the scientific community gravitates toward sustainable methodologies,^18−21^ cellulose emerges as a promising material. However, despite its broad consideration in the biomedical field, there remains a notable gap in the literature regarding in-depth *in vivo* analyses of different cellulose forms, especially those derived from diverse sources and processing methods. These issues are crucial in evaluating cellulose’s potential to replace PP meshes in biomedical applications.

Bacterial nanocellulose (BNC), regenerated cellulose (RC), and cellulose nanofibrils (CNFs) represent distinct cellulose structures, each derived from different sources and production methods, and therefore, exhibit inherently different properties. BNC, produced by specific bacterial genera such as *Komagataeibacter*,^1,21,22^ forms nanofibril networks, which are promising for their biocompatibility, moisture retention, and suitability for biomedical applications, including skin, bone, and vascular grafts,^23−25^ as well as wound dressings.^26−28^

In contrast, RC is traditionally sourced from wood fibers,^29^ agricultural^30,31^ or forestry streams,^32^ which are dissolved and then regenerated.^2,33−38^ RC is commonly used in the textile industry, where it is valued for its mechanical durability in the form of filaments, films and wearables.^1^ Lastly, CNF, extracted from plant cell walls, offers high mechanical strength and a high surface area due to its high level of deconstruction.^16,17^ This makes CNF a suitable candidate for creating customizable structures with adjustable shape, porosity, and microstructure.^39−44^

To the best of our knowledge, no *in vivo* biocompatibility studies have been published for alkali-dissolved (NaOH/H_2_O, ZnO) RC, nor has there been an analysis comparing the main features of BNC, RC, CNF, and PP under the same conditions. Therefore, we assessed the *in vivo* biocompatibility of cellulose-derived meshes using a rat subcutaneous implantation model with 80 healthy female Sprague–Dawley rats.^45^ Acknowledging the limitations in achieving identical material structures across the tested cellulose forms (e.g., geometry, porosity, density), we aimed to test the hypothesis that BNC, RC, and CNF—due to their different sources, production methods, and resulting unique properties—could potentially replace PP, introducing sustainable, versatile, and biocompatible meshes for biomedical applications.

## Materials and Methods

### Materials

**Figure 1:**
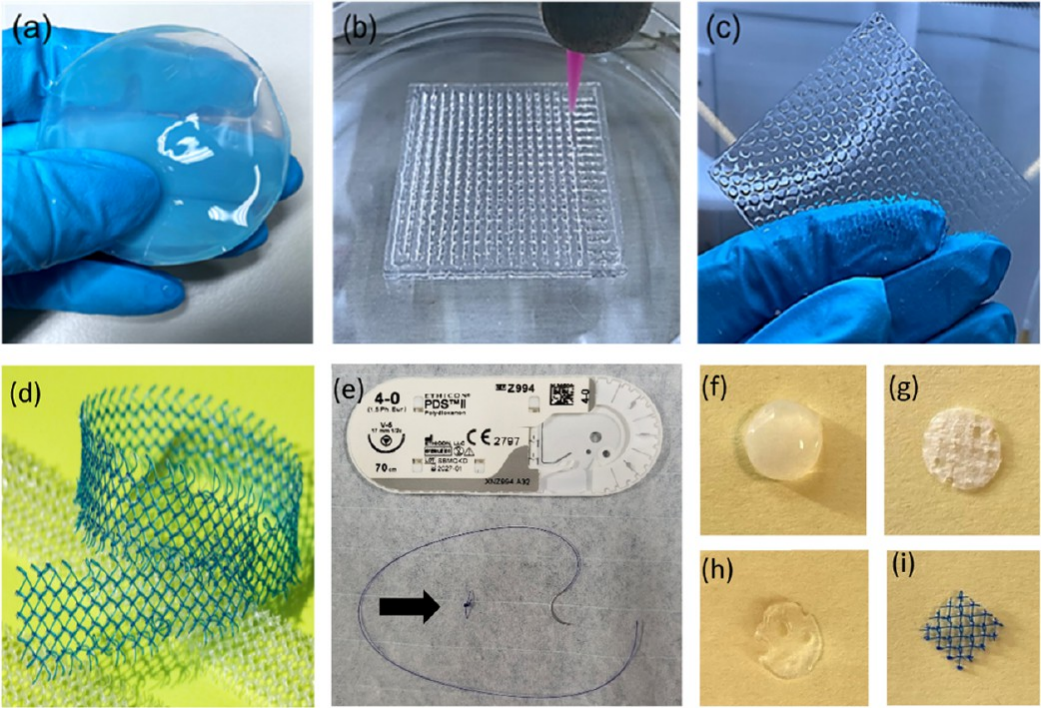
Cellulose-derived samples in wet condition: (a) bacterial nanocellulose (BNC), (b) 3D-printed cellulose nanofibrils (CNF), (c) regenerated cellulose (RC). (d) Commercial polypropylene (PP) based mesh (Photograph by Kalle Kataila, Aalto University). (e) Polydioxanone (PDS, Johnson & Johnson, New Brunswick, NJ). Standardized 8 mm samples: (f) BNC, (g) 3D-CNF, (h) RC, (i) PP. *Black arrow* = PDS suture.

Figure 1 displays images of all samples tested in this study before cutting. They included commercial PP meshes, implants made from different cellulose forms and polydioxanone (PDO) used as a sham procedure.

#### Polypropylene (PP)

Gynecare TVT Exact PP mesh was obtained in a sterile package from Johnson & Johnson (New Brunswick, NJ). The mesh was cut, autoclaved (at 121 °C for 15 min), and utilized as control samples for *in vivo* tests (Figure 1d,i).

#### Bacterial Nanocellulose (BNC)

The strain used for BNC production, *Komagataeibacter medellinensis*, was provided by the School of Engineering, Universidad Pontificia Bolivariana, Colombia.^46^ D-(+)-glucose, sodium phosphate dibasic (Na_2_HPO_4_), peptone, yeast extract, citric acid, sodium bromide, sodium hypochlorite, and sodium hydroxide were purchased from Sigma-Aldrich (St. Louis, MO). Milli-Q water (purified using a Millipore Synergy UV unit, Burlington, MA) was used throughout the experiments (18.2 MΩ cm).

First, glucose, yeast extract, peptone, and Na_2_HPO_4_ were mixed in a dry mass ratio of 8:2:2:1. Milli-Q water was then added to achieve a final volume of 1 L, containing 20 g of glucose, 5 g of yeast extract, 5 g of peptone, and 2.5 g of Na_2_HPO_4_. All components were fully dissolved, and the pH of the medium was adjusted to 4.5 with citric acid. The container was sterilized in an autoclave at 121 °C for 15 min and then cooled to room temperature. The bacterial strain was added to the culture medium and gently shaken to ensure homogeneity. The BNC culture medium was poured into sterilized containers and incubated at 28 °C for 10 days.

The resulting BNC biofilm was washed several times with deionized water and left in it for 24 h, with the water changed several times daily to remove residual components from the growth medium. The bacterial cellulose pellicle was then purified with 0.1 M NaOH at 60 °C for 4 h, followed by several washes in hot deionized water for 6 h.^47^ Finally, the BNC was stored in autoclaved Milli-Q water at 4 °C.

#### Regenerated Cellulose (RC)

The cellulose source used was Avicel PH-101 microcrystalline cellulose (50 μm particle size, degree of polymerization 400), purchased from Sigma-Aldrich, Ireland, and used without modifications. Sodium hydroxide (NaOH, purity 99.6%) from VWR Chemicals and zinc oxide (ZnO, pro-analysis grade) from Sigma-Aldrich were used to prepare the cellulose solvent. Sulfuric acid (H_2_SO_4_) and sodium sulfate (Na_2_SO_4_), both reagent grade from Sigma-Aldrich were used to prepare the regeneration bath. All solutions were prepared using Milli-Q water (Millipore Corporation, resistivity 18 MΩ·cm).

Cellulose (7% w/w), previously dried under vacuum (200 mbar, 60 °C, 12 h), was dissolved in a 2.3 M NaOH solution with the addition of ZnO, maintaining a ZnO/NaOH mass ratio of 0.167. This cellulose was added to the precooled NaOH/ZnO aqueous solution at −5 °C. The mixture was stirred at 300–700 rpm in a vessel with a cooling jacket using a water/propylene glycol 1:1 mixture, maintaining a temperature of −5 °C for 24 h. After stirring, an opaque and viscous solution was obtained, which was then frozen at −17 °C for 12 h. As reported,^38^ this freezing step enhances the interaction between NaOH hydrated shells and the reactive hydroxyl groups of cellulose, facilitating dissolution. Upon thawing,^19^ a transparent and fully dissolved ink was obtained.

The cellulose ink was used immediately after thawing to produce the RC films using ink extrusion with a nozzle gauge and infill density that were carefully controlled. Extrusion was performed using a 250 μm diameter nozzle at an extrusion pressure ranging from 6–15 kPa and printing speed of 11 mm/s. The films were extruded on glass Petri dishes to obtain samples of 1 mm thickness with a 75% infill density. This process involved repeated injections of the RC ink along the horizontal plane, leading to merging and consolidation of individual printed lines into a single, uniform surface. The printed film patches were coagulated for 1 h in a coagulation solvent composed of 10% w/w H_2_SO_4_ and 10% w/w Na_2_SO_4_, washed with Milli-Q water until achieving a stable pH, autoclaved, and stored in Milli-Q water at 4 °C.

The use of low-viscosity RC inks, with cellulose concentration below 12 wt % in alkali, allowed to produce smooth and even films at room temperature. As demonstrated in our previous studies,^17^ these low-concentration inks are ideal for creating uniform films showing no visible surface textures.

#### 3D-Printed Cellulose Nanofibrils (CNFs)

TEMPO-oxidized cellulose nanofibrils (TOCNF) were produced using TEMPO-mediated oxidation, followed by the disintegration of never-dried, fines-free, and fully bleached hardwood (birch) fibers sourced from a Finnish pulp mill, as described previously.^43^ The cellulose fibers were immersed in Milli-Q water with the addition of 0.013 mmol/g TEMPO and 0.13 mmol/g sodium bromide. Subsequently, 5 mmol/g sodium hypochlorite was added, and the pH was adjusted to 10 using 0.1 M sodium hydroxide. The mixture was maintained at room temperature and stirred for approximately 6 h. The resulting fibers were washed several times with deionized water until a neutral pH was achieved. The fibers were then fibrillated using a microfluidizer (M-110P, Microfluidics Inc., Newton, MA) at a pressure of 1400 bar with a single pass.

A BIOX bioprinter from CELLINK (Sweden) equipped with pneumatic multiprintheads was used to 3D print lattice structures, as shown in Figure 1b. The TOCNF was placed into 3 mL clear pneumatic syringes and extruded through a 22-gauge (410 μm diameter) sterile blunt needle. Lattice structures were printed on a 100 mm diameter glass Petri dish, subjected to UV-sterilization and autoclaving at 121 °C for 15 min following freeze-drying to ensure complete sterilization.

#### Polydioxanone (PDO)

A monofilament, slowly resorbable suture^48^ (PDS, Johnson & Johnson, New Brunswick, NJ) was used as a sham procedure (Figure 1e).

#### Design Criteria

All implants were standardized to a uniform size of 8 mm diameter. Circular samples were cut from cellulose-derived meshes using a sterile biopsy punch, and square samples were cut from PP (Figure 1f–i). The CNF and RC implants were 3D-printed with similar thickness and infill density to replicate the structure of the PP-based control mesh. Although the overall geometries were consistent, small variations in the viscosity of RC and CNF inks resulted in differences in interlayer adhesion and void coalescence, subsequently leading to small square infill patterns that merged and round out into circular features.

#### Material Characterization

The characterizations of each material were thoroughly examined by the authors in previous publications.^47,49,50^ All cellulose-derived materials used in the study have similar tensile strength and high wettability, measured by the water contact angles (less than 90°).^51^ RC films presented an elastic modulus ranging from 4.5 to 8 GPa and a tensile strength of 120–170 MPa.^49^ Those for CNF and BNC corresponded to 10 and 10–18 GPa for the elastic modulus and 200–300, and 200–250 MPa for the tensile strength, respectively.^47,52^

TEMPO-oxidized CNF has a surface charge of about 1.36 mmol_COOH_/g,^53^ The introduction of carboxyl groups increases hydrophilicity and enhances cell adhesion, proliferation, and compatibility with host tissue.^54^ Indeed, hydrophilic surfaces generally promote better interaction with cells and reduce adverse immune responses.^17^ Based on XRD, FTIR, and GPC analyses,^44,47,49,53^ RC is primarily composed of cellulose type I while the CNF and BNC cellulose correspond to the cellulose polymorph. For BNC, trace amounts of proteins were also detected, which is not expected to impact the materials′ performance *in vivo*.^47^

### Methods

#### Scanning Electron Microscopy (SEM)

The microstructure of the surface and cross-section of implants were observed using a scanning electron microscope (Carl Zeiss AG, Oberkochen, Germany) operated at an accelerating voltage of 2 kV. Samples were fixed on metal stubs with double-sided carbon tape and sputter-coated with a 3–4 nm layer of gold–palladium alloy using a LEICA EM ACE600 (Leica Camera AG, Wetzlar, Germany) sputter coater.

#### Animal Tests

A total of 80 healthy female Sprague–Dawley rats, aged 10 to 12 weeks, were used in this study. Based on previous literature^55,56^ and the principles of the 3Rs,^57^ a sample size of 10 animals per group was calculated to achieve 80% power to detect an effect size of 1 with a standard deviation of 0.8 in the histological scoring of the inflammatory host response between groups, using a semiquantitative scoring system.^58^ An α level of 0.05 was assumed, and group comparisons were performed using the Wilcoxon signed-rank test. The rats were housed in pairs under equal conditions with free access to food, water, and environmental stimuli. They were acclimatized for 1 week before the study and provided with weekly access to a large activity cage during the follow-up period as part of the refinement. The experimental design was approved by the National Animal Ethics Committee (ESAVI/4488/2021). Animal housing adhered to the European Directive (Directive 2010/63/EU) and Finnish legislation (497/2013).

##### Procedures

Each rat was implanted with two subcutaneous implants: PP as a control on the left side of the spine, and either BNC, RC, CNF or PDO (PDS, Johnson & Johnson, New Brunswick, NJ) as a sham procedure, on the right side (Table 1). Prior to induction of anesthesia, the rats were weighed and premedicated with buprenorphine. Anesthesia was induced in an induction chamber with 4.5% isoflurane and 600 mL/min air flow. Upon loss of consciousness, the rat was transferred to a mask with 2–2.3% isoflurane and 600 mL/min air flow, then positioned ventrally on a heated plate (37 °C). The back was shaved and disinfected with 80% alcohol, following standard aseptic protocols.

**Table 1:** Experimental Groups in a Rat Subcutaneous Transplantation Model

**group**	**animals/group**	i**mplant left**	**implant right**	**suture material**^a^
1	20	PP^b^	BNC^c^	4–0 PDO^d^
2	20	PP^b^	RC^e^	4–0 PDO^d^
3	20	PP^b^	CNF^f^	no
4	20	PP^b^	SHAM^d^	4–0 PDO^d^

a Implants were secured to the underlying tissue with one suture.

b Polypropylene (PP).

c Bacterial nanocellulose (BNC).

d Polydioxanone (PDS, Johnson&Johnson, New Brunswick, NJ).

e Regenerated cellulose (RC).

f 3D-printed cellulose nanofibrils (CNFs).

**Figure 2:**
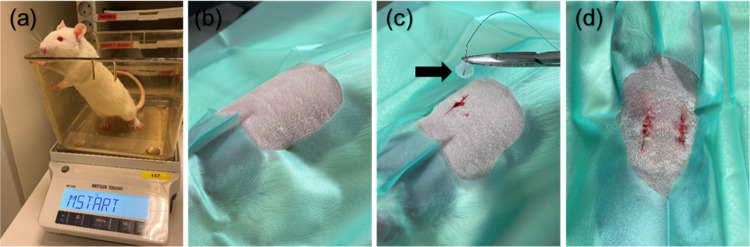
Implantation procedure. (a) Weighing of a rat prior to anesthesia. (b) An anesthetized rat at ventral recumbency on a heated plate (37 °C) with back shaved and disinfected with 80% alcohol. (c) Two vertical incisions were made on each side of the spine caudal to the scapulae, a subcutaneous pocket created by blunt dissection, and an implant (bacterial nanocellulose, BNC) secured to the underlying tissue. (d) Routine skin closure with simple interrupted sutures. *Black arrow* = BNC implant.

Two vertical incisions (1–1.5 cm) were made through the skin, 2–3 cm on each side of the spine caudal to the scapulae, and subcutaneous pockets were created by blunt dissection. Each mesh, except for the fragile CNF, was secured to the underlying tissue with one suture of a resorbable, monofilament suture (4–0 PDO, PDS, Johnson & Johnson, New Brunswick, NJ) to prevent implant migration and ease identification at excision (Table 1). The skin was closed with simple interrupted 4–0 poliglecaprone sutures (Monocryl, Johnson & Johnson, New Brunswick, NJ) (Figure 2). Each rat was uniquely marked for individual identification using a specific combination of earmarks made by an ear punch.

After surgery, the rats were monitored and kept on a 37 °C plate until they fully recovered. For pain relief, they received subcutaneous injections of carprofen (Rimadyl, Zoetis Animal Health, Denmark), a nonsteroidal anti-inflammatory drug, administered before surgery and continued for 2 days postoperatively. The rats were checked twice daily for the first 7 days after surgery, and subsequently, once daily until euthanasia. General behavior and movement were assessed on a scale of 0–2 adapted from Carstens & Moberg:^59^ 0 indicating normal behavior and movement, 1 indicating ungroomed appearance, partial piloerection, hyporexia, or abnormal stance, and 2 indicating severe piloerection, anorexia, or recumbency. Pain levels were evaluated using the Rat Grimace Scale,^60^ also on a scale of 0–2. Wounds were evaluated on a scale of 0–5^61^ and photographed on days 1, 3, 5, 7, 30, and 90.

##### Follow-Up, Macroscopic Evaluation, and Tissue Collection

Follow-up assessments were conducted at one and three months, with ten samples of each material evaluated at each time point. At the conclusion of the follow-up period, the rats were euthanized using CO_2_ asphyxiation, followed by decapitation. After euthanasia, the scar and surrounding tissue were assessed for local inflammation, infection, seroma, or abscess formation, graded on a scale of 0 to 1 (0 = absent, 1 = present).

The implanted mesh, or PDO as a sham procedure, was excised with 3 mm margins, including skin, subcutaneous tissue, and muscle. Tissue samples were fixed in 4% paraformaldehyde and processed for paraffin embedding using an automated tissue processor (Tissue Tek VIP, Sakura Finetek, Torrance, CA). Samples were then sectioned longitudinally into 5 μm slices with a microtome (Microm cool-cut HM 355S, Thermo Scientific, Kalamazoo, MI) for histological analysis. The sections were stained with hematoxylin and eosin (H&E) following standard procedures,^62^ and assigned coded identification numbers.

##### Histopathology

Histological assessment was conducted blindly by our experienced pathologist (H.S. author) and a veterinary surgeon (N.M.M.P. author). A customized scale adapted from a semiquantitative scoring system (EN ISO 10993-6 Annex E)^58^ was developed to differentiate the degree of various host tissue responses. The biocompatibility of the implanted mesh materials was assessed by analyzing the host inflammatory response, including cellularity, foreign body reaction (FBR), and cell ingrowth. The integral classification score comprised the presence and for foreign body reaction, the number of different cell types per unit area within the implanted mesh and the tissue-implant interface. Specific evaluations included:

- **Foreign Body Reaction (FBR):** Presence of macrophages and foreign body giant cells (FBGCs) (Table 2).
- **Acute Inflammation:** Presence of neutrophils.
- **Chronic Inflammation:** Presence of lymphocytes.
- **Granulation Tissue:** Presence of plasma cells, sporadic lymphocytes, and granulocytes.
- **Scar Formation:** Presence of fibrosis.

**Table 2:** Foreign Body Reaction to an Implant Adapted from EN ISO 10993-6 Annex E^58^

**score**					
**cell**type/response	**0**	**1**	**2**	**3**	**4**
**foreign body reaction**					
macrophages	0	1–40/hpf^a^	40–80/hpf^a^	heavy infiltrate, 80–180/hpf^a^	packed, >180/hpf^a^
foreign body giant cells	0	1–2/hpf^a^	3–5/hpf^a^	5–10/hpf^a^	sheets, >10/hpf^a^

a Abbreviations: high-power (400×) field.

Granulation tissue, characterized by proliferating capillaries and variable inflammatory reactions, was noted to regress and transform into scar tissue with reduced cellularity. These histological characteristics were evaluated in at least three sections per tissue sample and double-counted. Scoring was performed on a scale from 0 to 4 for FBR (0 = absent, 1 = mild, 2 = moderate, 3 = heavy infiltrate, 4 = packed)^58^ (Table 2) and from 0 to 1 for acute and chronic inflammation, granulation tissue, and scar formation (0 = absent, 1 = present). The thickness of fibrosis was evaluated as recommended by EN ISO 10993–6 Annex E^58^ (0 = absent, 1 = narrow band = 1–2 cell layers in thickness, 2 = moderately thick band = < 10 cell layers in thickness, 3 = thick, contiguous band along length of tissue, 4 = extensive, thick zone with effacement of local architecture).

### Statistical Analysis

A proportional-odds cumulative logit model was employed, utilizing mesh type, time point, and their interaction as fixed factors, to compare the severity of tissue reactions among different explants. This model assessed the probability of higher severity in tissue reactions. Odds ratios with 95% confidence intervals were computed to quantify differences relative to the PP mesh.

To further evaluate tissue reaction severity within individual rats across different meshes (or sham for PDO), within-rat differences were calculated compared to the PP mesh. Descriptive tabulation of these differences was initially performed by mesh type (or sham), followed by formal analysis using a cumulative logit model with mesh as the sole fixed factor. Pairwise odds ratios derived from this model facilitated comparisons between meshes. Statistical significance was defined as *p* < 0.05. All analyses were conducted using SAS software, version 9.4 (SAS Institute Inc., Cary, NC).

## Results and Discussion

### PP and RC Meshes Are Less Porous than Those Made from BNC and CNF

**Figure 3:**
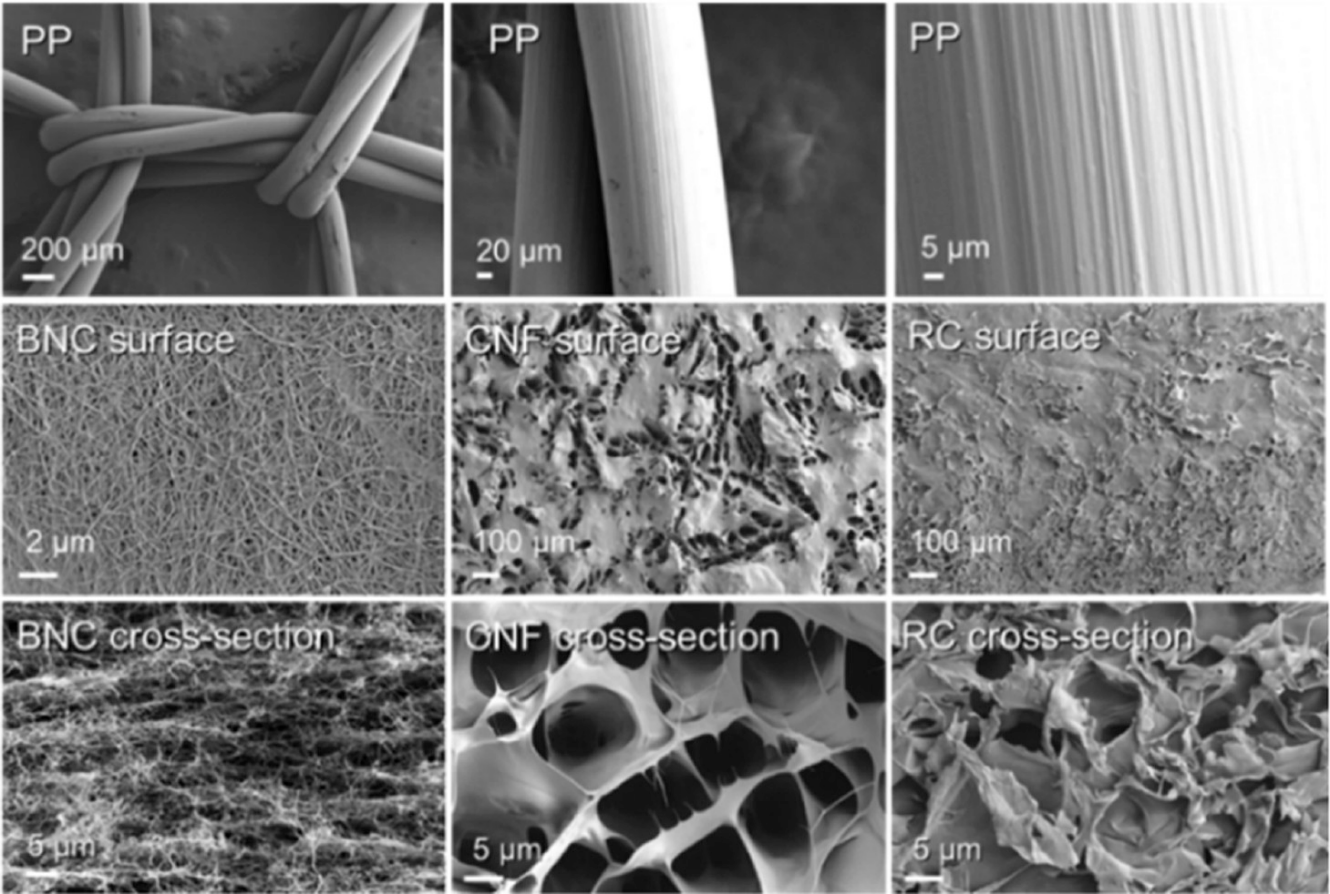
Scanning electron microscopy (SEM) of the meshes preimplantation. The surface and cross-section of polypropylene (PP), bacterial nanocellulose (BNC), 3D-printed cellulose nanofibrils (CNFs), and regenerated cellulose (RC).

Surface and cross-sectional analyses of the meshes were conducted using SEM prior to implantation to investigate their porosity, morphology, and microstructural differences. The porosity of the solid phase of the cellulose-derived meshes in cross-section SEM images indicated the highest porosity for CNF and the smallest for RC (Figure 3). BNC, characterized by physical entanglement, exhibited a relatively dense surface network while displaying a highly porous layered cross-section. These findings align with previous studies on fibrillated cellulose.^16^

CNF showed larger openings and pores on both its surface and cross-section, resulting in a rougher texture compared to BNC, RC, and PP. Previous research on porous nanocelluloses has indicated that myoblast cells tend to attach less effectively to surfaces with higher roughness.^44^ However, attachment behavior can vary significantly depending on factors such as cell type and surface charge. In contrast, RC, following regeneration and coagulation, exhibited fewer pores and formed a more compact microstructure that resembled the homogeneous, smooth surface characteristic of PP (Figure 3).

In addition to considering the porosity of the solid fraction of the meshes, another crucial factor is the open area of the structure. The PP mesh consisted of a grid of solid, nonporous filaments arranged in a woven structure with large open areas (Figure 1d). In its wet state, BNC forms a highly microporous membrane (Figure 1a), similar to RC films (Figure 1c). The cellulose meshes, created through direct ink writing of cellulose nanofibrils, exhibit a grid-like structure characterized by smaller square-like open areas. These filaments comprise the solid phase and possess inherent microporosity and permeability. The configuration of these open areas (square) on the mesh surface, along with the biomaterial’s topography, play a pivotal role in influencing the intensity of the host tissue’s response.^11,63^

Pore size is considered the most important characteristic of the solid phase of the material as it determines interactions with cells and potential integration.^64^ Cells and host tissues favor a porous microstructure due to enhanced transport of oxygen and nutrients.^64^ Differences in porosity allow cells to penetrate through the pores of BNC, RC, and CNF, unlike the solid microstructure of PP. The mesh configuration of CNF was intentionally designed to increase porosity and surface area. The open structure of the mesh allows for better cell adhesion, proliferation, and tissue integration, crucial for certain biomedical applications, such as wound healing.^43^

It is noteworthy that PP and CNF meshes were stored and implanted in a dry condition to ensure sterility. The wet strength of cellulose is generally lower than its dry strength.^50^ However, both BNC and RC retained a significant level of their strength in wet conditions.

The final performance of the materials in the host tissue depend not only on their porosity, but also their mechanical properties and chemical composition, surface charge, and wettability.^51^ All the cellulose-derived materials used in the study have similar tensile strengths^47,49,52^ and high wettability, and were expected to promote tissue interaction.^51^ The pore size and surface charge of the same materials can be adjusted to promote cell interactions. Bioinert nanomaterials can be rendered bioactive by adjusting the magnitude of their surface charge density. This adjustment can trigger interactions with oppositely charged proteins and ions from the surrounding biological environment, facilitating cell membrane interactions and enhancing cell adhesion and proliferation.^65^

The morphology of cellulose-derived materials is influenced by the source of cellulose and the synthesis processes used for creating meshes, patches, and other cellulosic materials. Cellulose regenerated through solvent dissolution typically exhibits smooth macroscale morphologies with minimal roughness.^50^ In contrast, CNF dispersed in water can be regenerated to mimic the morphology of RC while allowing for small surface roughness.^66^ In this study, both CNF and RC demonstrated smooth macroscale surfaces, although CNF, composed of smaller building blocks, potentially display higher accessibility for cell-material interactions.^67^ In the case of BNC, production through bacterial metabolism limits control over the microscale surface features.^68^ The specific methods selected for transforming BNC, CNF, and RC were chosen for their simplicity and energy efficiency. Therefore, comparisons should consider the factors mentioned above. Each system is discussed on its own merits regarding viable procedures for manufacturing meshes and patches.

While it is possible to tailor the morphology and macromolecular structure of cellulose to create structures with similar visual features—such as through mechanical modification or the fabrication of 3D-printed meshes—it is important to note that these processing steps can significantly impact the mechanical and microstructural characteristics of the final structures, as well as sterility.

### Implanted Cellulose-Derived Meshes Do Not Compromise Rat Well-Being Or Survival

Overall, 79 out of 80 implanted rats (98.8%) survived the study period and exhibited expected weight gain. One rat was euthanized prematurely 9 days postoperatively due to a tumor-like lesion on the right hind leg. The data from this rat was excluded from the study.

Of the 158 wounds assessed, 146 (92.4%) healed without complications (Figure S1a–e). The majority (83.3%) of wound reactions observed at 7 days and one month postimplantation were associated with wounds implanted with PP meshes (Figure S1f–h, Table 3) All wound reactions resolved either spontaneously or with local treatment. No wound reactions were noted at three months postimplantation.

**Table 3:** Wound Reactions in a Rat Subcutaneous Transplantation Model

**follow-up**	**wound reaction**	**BNC**^a^	**RC**^b^	**CNF**^c^	**SHAM**^d^	**PP**^e^	**total number of wound reactions**
7 days	mild erythema and swelling			1		6	7/158 (4.4%)
1 month	mild erythema and swelling					1	5/158 (3.2%)
	erythema and clear discharge		1			1	
	abscess					2	

a Bacterial nanocellulose.

b Regenerated cellulose.

c 3D-printed cellulose nanofibrils.

d Polydioxanone (PDS, Johnson&Johnson, New Brunswick, NJ).

e Polypropylene.

The higher incidence of wound reactions in wounds implanted with PP meshes compared to previous reports^15,69−71^ underscores the importance of consistent macroscopic evaluation of wounds, as conducted in this study.

Two abscesses (S1h, Table 3) were detected in the wounds implanted with PP, attributable to intraoperative aseptic issues, but did not affect the overall well-being or survival of the rats. These findings are consistent with previous *in vivo* studies involving subcutaneous implantation of BNC,^72,73^ and CNF,^39^ where no clinical signs of infection were reported at the implantation sites.

### BNC and RC Resist Degradation *In Vivo*

At explantation, BNC, RC, and PP meshes were easily retrieved and remained cohesive implying minimal degradation in 90 days. In contrast, CNF meshes were more challenging to visualize from host tissue, suggesting notable implant degradation and/or fragmentation. All implanted meshes integrated into the surrounding tissues, without any macroscopic signs of adverse reactions around the implanted sites.

The *in vitro* degradation profile of BNC was analyzed in a previous study.^49^ After 28 days of exposure to pH 7.4 and pH 5, BNC exhibited mass losses of less than 2 and 4%, respectively, primarily due to fibril detachment during the washing step and characterization, indicating its potential for long-term tissue support.^49^ Similarly, chitosan-modified TOCNF samples showed a maximum weight loss of about 10% after 28 days.^47^ For RC, the initial dissolution in alkali promotes gelation.^50^ The *in vitro* and *in vivo* degradation profiles are expected to differ due to the chemical, physical, and mechanical interactions, particularly related to host tissue morphology, integration with the host tissue, and mechanical tension during tissue healing. Based on previous studies,^43,47^ highly crystalline nanocellulose resists degradation and weight unless subjected to enzymatic, hydrolytic, or autocatalytic oxidation, which are not expected to occur *in vivo.*(43,47)

### All Implanted Materials Induced a Foreign Body Reaction in Host Tissue

A total of 126 samples from 79 rats were included in the analysis. Thirty-two samples were excluded from histopathological evaluation: 7 due to nonrepresentativeness and 25 due to technical processing issues (Table 4). For a detailed histopathological assessment, refer to Figure S2 in the Supporting Information.

**Table 4:** Cell Response to Different Implants in a Rat Subcutaneous Transplantation Model^a^

	**parameter**	**BNC**^b^	**RC**^c^	**CNF**^d^	**SHAM**^e^	**PP**^f^
follow-up 1 month						
**number of explants**		9	6	7	7	39
	**foreign body reaction**^g^					
	1	3 (33.3%)	3 (50%)	2 (28.6%)	3 (42.9%)	21 (53.8%)
	2	4 (44.4%)	1 (16.7%)	2 (28.6%)	2 (28.6%)	6 (15.4%)
	3	1 (11.1%)		1 (14.2%)		
	4					
	**acute inflammation**^h^		1 (16.7%)	1 (14.3%)	1 (14.3%)	4 (10.3%)
	**chronic inflammation**^i^					1 (2.6%)
	**granulation tissue**^j^	1 (11.1%)	1 (16.7%)	1 (14.3%)	2 (28.6%)	7 (17.9%)
	**scar**^k^		1 (16.7%)		1 (14.3%)	6 (15.4%)
	**excluded**^l^	1 (10%)	4 (40%)	3 (30%)	3 (30%)	1 (2.6%)

follow-up 3 months						
**number of explants**		7	7	6	8	30
	**foreign body reaction**^g^					
	1	4 (57.1%)	7 (100%)	1 (16.7%)	5 (62.5%)	11 (36.7%)
	2	2 (28.6%)		2 (33.3%)	2 (25%)	12 (40%)
	3	1 (14.3%)		2 (33.3%)		1 (3.3%)
	4			1 (16.7%)		
	**acute inflammation**^h^					
	**chronic inflammation**^i^					1 (3.3%)
	**granulation tissue**^j^					3 (10%)
	**scar**^k^				1 (12.5%)	3 (10%)
	**excluded**^l^	3 (30%)	3 (30%)	4 (40%)	2 (20%)	10 (25%)

a The numbers in the parameter column represent the severity of the cell response. Scores from 0 to 4 (0 = absent, 1 = mild, 2 = moderate, 3 = severe, 4 = packed) were used for evaluation of foreign body reaction^g^ and scores from 0 to 1 (0 = absent, 1 = present) for evaluation of the other cell responses. The numbers in the treatment columns represent the number of samples where some degree of cell response was observed.

b Bacterial nanocellulose.

c Regenerated cellulose.

d 3D-printed cellulose nanofibrils.

e Polydioxanone (PDS, Johnson&Johnson, New Brunswick, NJ).

f Polypropylene.

g Giant cells and macrophages.

h Neutrophils.

i Lymphocytes.

j Plasma cells, sporadic lymphocytes, and granulocytes.

k Connective tissue.

l Technical issues, nonrepresentative samples, premature euthanasia due to study-unrelated cause.

#### Bacterial Nanocellulose (BNC)

**Figure 4:**
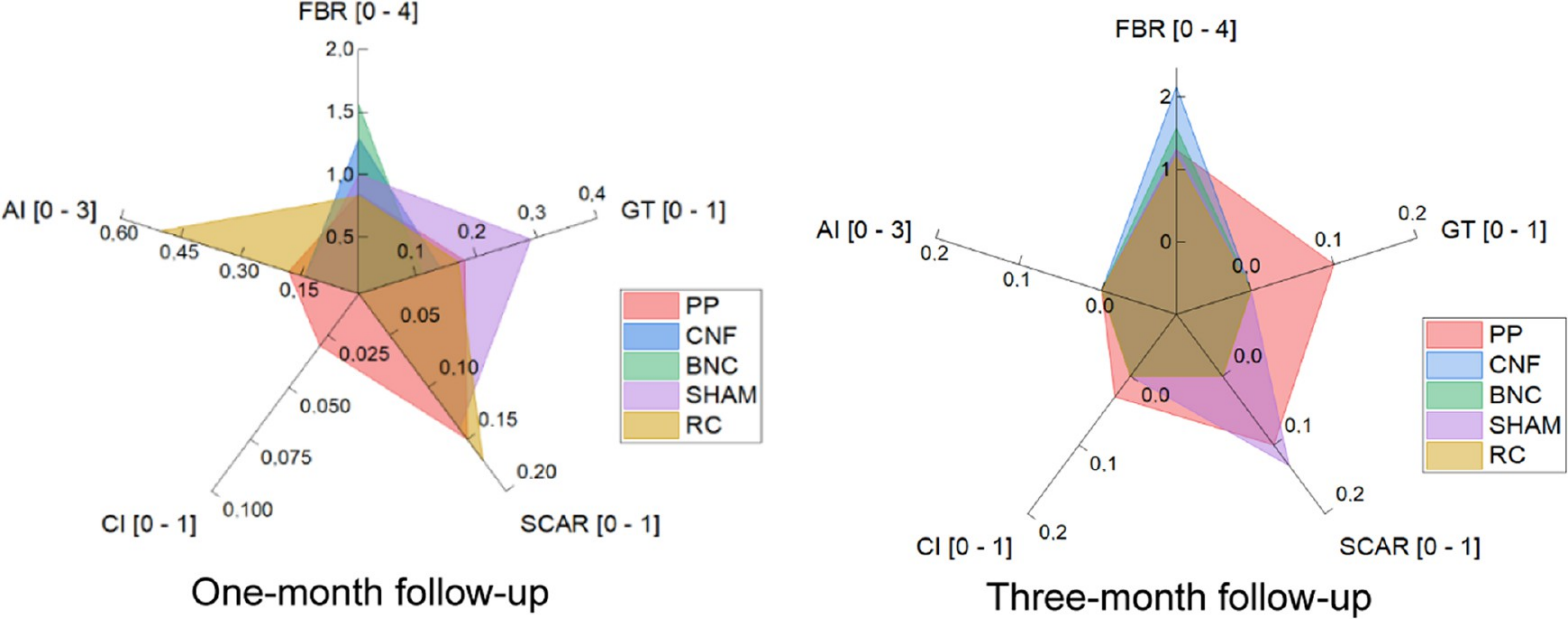
Cell response to bacterial nanocellulose (BNC), regenerated cellulose (RC), 3D-printed cellulose nanofibrils (CNFs), polydioxanone (PDS, Johnson&Johnson, New Brunswick, NJ) as sham procedure, and polypropylene (PP) in a rat subcutaneous transplantation model at two different time points. FBR = foreign body reaction, GT = granulation tissue, AI = acute inflammation, CI = chronic inflammation.

The majority (8/9, 88.9%) of implanted BNC meshes exhibited mild-to-severe FBR at one-month postimplantation, with all samples (7/7, 100%) showing similar reactions at three months. At one month, the FBR in BNC implants was significantly more severe compared to the PP meshes (*p* = 0.0150, OR 6.02, 95% CI 1.43–25.36). However, by comparing one- and three-month follow-ups, the difference in FBR diminished (*p* = 0.482, OR 1.74, 95% CI 0.37–8.26) (Table 4, Figure 4). This observed inflammatory response aligns with findings from our recent *in vitro* study, where BNC induced monocyte activation but suppressed the proinflammatory macrophage-like phenotype induced by 12-O-Tetradecanoylphorbol-13-acetate (TPA).^47^

Our results are consistent with previous *in vivo* studies on BNC implantation, such as intradermal implantation in rabbits for 28 days^74^ and subcutaneous implantation in sheep for 1–32 weeks,^75^ which showed a chronic phase of inflammation with foreign body giant cells (FBGCs). In contrast, studies by Pértile et al.^72^ and Helenius et al.^73^ did not report FBR in rats implanted with BNC subcutaneously for periods ranging from 3 to 12 months. Differences noted may be attributed to variations in material characteristics (shape, size, porosity) and animal models used.^76^ The porous nature of BNC facilitates cell migration and predisposes to inflammatory reactions in host tissue. While the BNC samples used in our study were washed with NaOH (aq.), residual traces cannot be completely ruled out.

#### Regenerated Cellulose (RC)

The majority (4/6, 66.7%) of the implanted RC meshes exhibited a mild-to-moderate FBR at one month postimplantation, while a mild FBR was observed in all samples (7/7, 100%) at three months. The severity of FBR in the RC meshes was notably similar to that observed with PDO and PP, displaying a mostly mild-to-moderate FBR at one-month postimplantation and a slightly milder FBR at three months (Table 4, Figure 4). This similarity in host tissue reaction can be attributed to the analogous microstructure of RC and PP meshes. Our results align with previously published studies,^77,78^ where PP mesh induced a mild but persistent FBR both as an intraperitoneal mesh in rats^77^ and in pelvic reconstructive surgery in humans.^78^

#### 3D-Printed Cellulose Nanofibrils (CNFs)

A mild-to-severe FBR was observed in the majority (5/7, 71.4%) of CNF meshes at one-month postimplantation. By three months, all CNF samples exhibited a moderate-to-severe or packed FBR (Table 4, Figure 4). Unlike RC meshes, where the initial FBR diminished over time, the FBR in CNF meshes intensified, with a significantly greater severity at three months (*p* = 0.0030, OR 19.14, 2.78–131.83) compared to PP.

In an *in vitro* study, Ajdary et al.^43^ demonstrated the high biocompatibility of 3D-printed TOCNF-based patches, finding that drug loading supported cardiac cell proliferation for 28 days. Although the number of samples in our study was limited, the FBR observed was more severe than in previous *in vivo* biocompatibility studies of TOCNF.^39−41^ Outstanding biocompatibility and wound healing efficacy of TOCNF combined with gelatin and aminated silver nanoparticles (Ag-NH2NPs) were reported in a 14-day *in vivo* wound healing study with mice.^41^ No FBR was elicited in a subcutaneous injection of TOCNF in a 12-week rat model.^39^ Furthermore, a subcutaneous rat transplantation study of a 3D aerogel blend of CNF and gelatin over 8 weeks showed significant improvement in FBGC reaction and acute inflammation between four and 8 weeks postimplantation.^40^

The CNF meshes were not easily identified at the three-month follow-up. The challenging visualization and the more severe FBR relative to PP were likely attributable to the mesh’s morphology and surface chemistry when implanted in a dry condition. Degradation depends on both the implant’s morphology and surface chemistry, as well as the mode of preparation.^39^ Mesh construction and composition appear to be more crucial in determining FBR after implantation than merely the reduction of the material itself.^5^ The rough surface and fragmentation of CNF might contribute to the development of a pronounced late FBR.^76^ Previous studies indicate that myoblast cells exhibit reduced attachment to surfaces with higher roughness;^44^ however, cell attachment behavior is highly influenced by the specific cell type and the overall surface charge.

No significant difference was detected when comparing the severity of FBR of BNC, RC, CNF, and PDO with PP within individual rats. The FBR detected in our study is consistent with the expected host tissue reaction to a foreign material. Implantation of a biomaterial typically initiates an inflammatory process aimed at preventing tissue damage, isolating and destroying the foreign material, and initiating the repair process. The acute inflammatory reaction generally subsides within a week, whereas a chronic inflammatory response can last for up to 4 weeks, leading to a granulation phase, including FBR.^4,78^

### Cellulose-Derived Meshes Induce Cell Ingrowth to Implanted Materials

**Figure 5:**
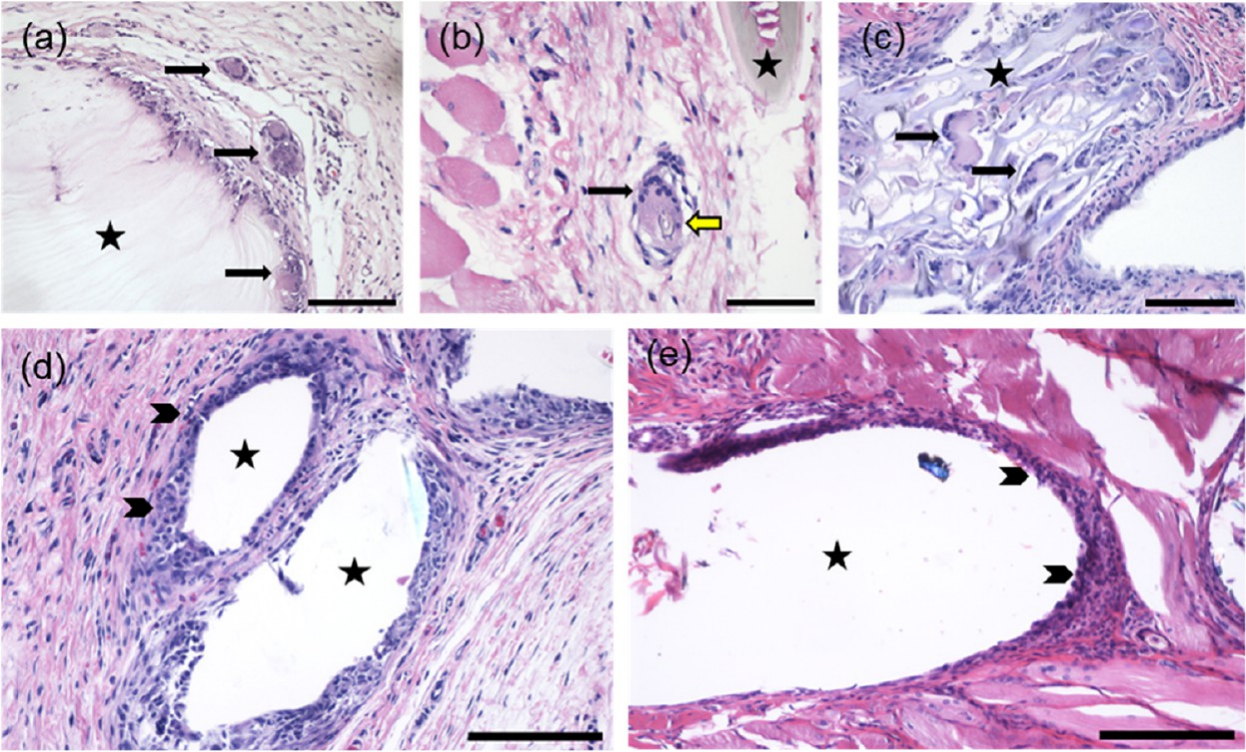
Histopathological analysis of different implants at day 30. (a) Foreign body giant cells in the border zone surrounding bacterial nanocellulose (BNC). (b) A foreign body giant cell phagocytosing regenerated cellulose (RC). (c) Biomaterial fragmentation and foreign body giant cell infiltration into 3D-printed nanofibrils (CNFs). (d) Macrophages in the border zone surrounding polypropylene (PP) and (e) polydioxanone (PDS, NJ). *Black arrow* = foreign body giant cell, *yellow arrow* = phagocytosed biomaterial, *arrowhead* = macrophages, *asterisk* = implanted mesh/mesh hole or, in case of sham, suture material. H&E staining. Bars 200 μm (a, c–e) and 100 μm (b).

**Figure 6:**
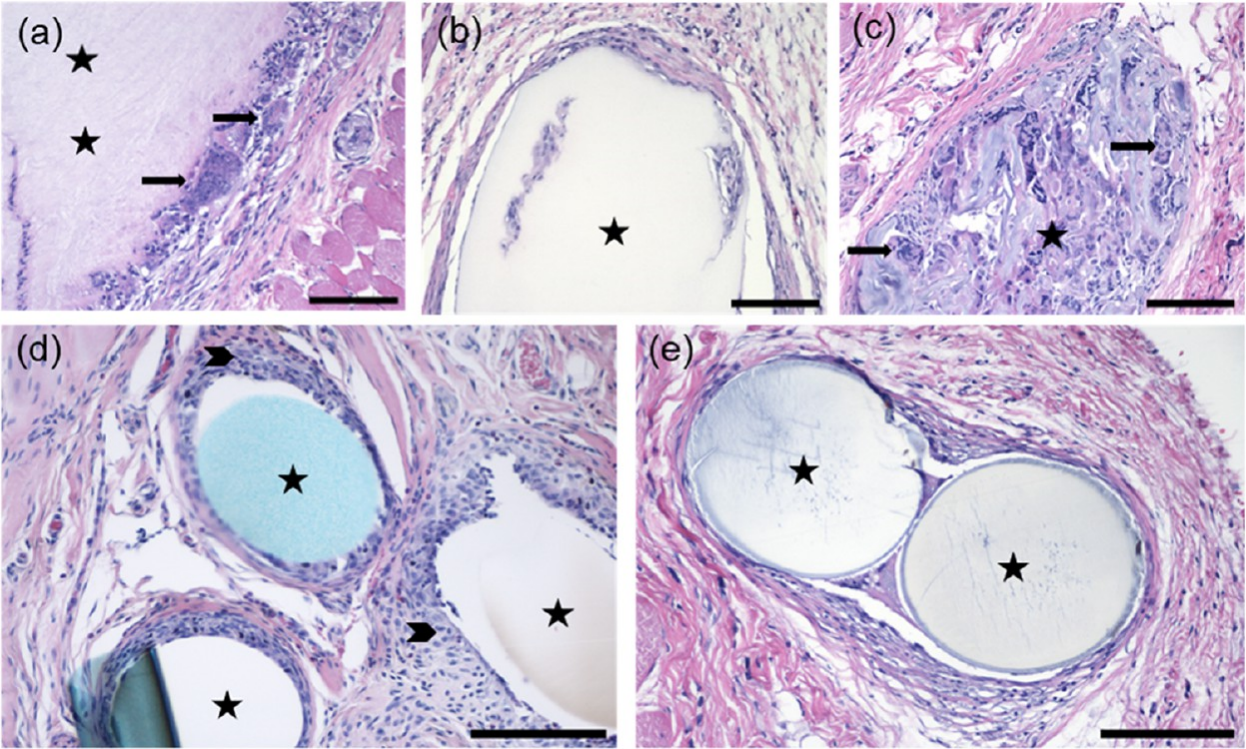
Histopathological analysis of different implants at day 90. (a) Foreign body giant cells in the border zone surrounding bacterial nanocellulose (BNC). (b) Macrophages in the border zone surrounding and infiltrating into regenerated cellulose (RC). (c) Biomaterial fragmentation and foreign body giant cell infiltration into 3D-printed nanofibrils (CNFs). (d) Macrophages in the border zone surrounding polypropylene (PP) and (e) polydioxanone (PDS, Johnson&Johnson, New Brunswick, NJ). *Black arrow* = foreign body giant cell, *arrowhead* = macrophages, *asterisk* = implanted mesh/mesh hole or, in case of sham, suture material. H&E staining. Bars 200 μm.

With BNC, RC, and CNF, the FBR was observed not only at the implant periphery, but also penetrating the material (Figures 5 and 6). Cell ingrowth was multifocally present at the tissue-implant interface, with some FBGCs engulfing the biomaterial (Figure 5b). Dense cell ingrowth, phagocytosis of the implanted material and implant fragmentation were consistently more extensive in CNF meshes, displaying a more indistinct border zone between the material and the surrounding tissues compared to the other explants at given time points (Figures 5c and 6c). Over time, integration with the host tissue was observed. Cellular penetration is expected to be more intense through porous implants, in agreement with Pèrtile^72^ and Helenius et al.^73^ In contrast, no cellular infiltration or fragmentation of the implanted material was observed with PP and PDO (Figures 5 and 6).

Macrophages are considered to be the most important cellular mediators of FBRs in biodegradable materials. When macrophages are not effective in removing the foreign material, they fuse into FBGCs.^63,79^ FBGCs persist in the tissues as long as the biomaterial is detected, eventually leading to degradation of the implanted material.^4,64^ A successfully implanted biocompatible biomaterial will integrate with the host tissue,^79^ as shown in the present study with BNC, RC, and CNF (Figures 5 and 6).

**Figure 7:**
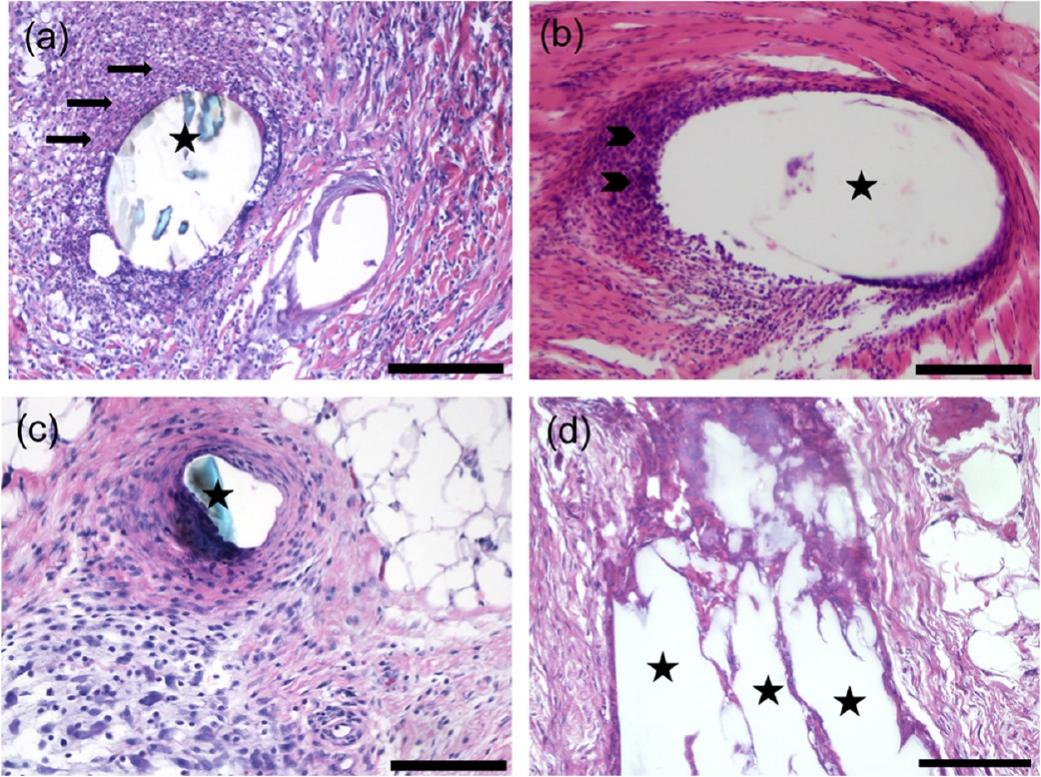
Histopathological analysis of different cell reactions surrounding implants with the exception of foreign body reaction. (a) Acute inflammation, day 30, polypropylene (PP). (b) Chronic inflammation, day 90, polypropylene (PP). (c) Granulation tissue with variable inflammatory reaction, day 90, polypropylene (PP). (d) Scar tissue, day 30, regenerated cellulose (RC). *Black arrow* = neutrophils, *arrowhead* = lymphocytes, and *asterisk* = implanted mesh/mesh hole. H&E staining. Bars: 200 μm in (a, c, d) and 400 μm in (b).

None of the implanted materials induced signs of necrosis, extensive fibrosis, or marked inflammatory reactions. Localized collections of neutrophils were detected in a total of 7/126 samples (5.6%) (Table 3, Figure 7a) either around the implant (3/7, 42%), or distant from the implantation site in subcutaneous tissue (4/7, 58%) indicating intraoperative aseptic failure. No signs of acute inflammation were observed in any of the samples three months postimplantation. In addition, no signs of chronic inflammation were observed in any of the cellulose-derived samples nor PDO in the given time points. Mild neovascularization with minimal capillary proliferation and focal, 1–3 buds of vessels; mild fatty infiltrate around the implants, and sporadic fibrocytes were generally displayed. Scar formation with mild to moderate fibrosis was observed in a total of 11/126 samples (8.7%) (Table 3, Figure 7d). Importantly, no extensive fibrosis, nor signs of necrosis were detected. No significant differences were observed when comparing acute and chronic inflammation, granulation tissue, and scar formation between BNC, RC, CNF and PDO implants and PP, nor between the implants within the same individual.

### Biocompatibility of BNC, RC, and CNF Meshes and Their Potential To Replace PP Meshes

An appropriate host response and active cell ingrowth were detected in all implanted cellulose-derived meshes in the host tissue. The presence of macrophages and FBGCs was not indicative of an adverse FBR. No signs of necrosis, extensive fibrosis, or marked inflammatory reactions were observed in any of the tissue samples. Implants reached a steady state of tolerance within the surrounding host tissue, suggesting that BNC, RC, and CNF meshes exhibit potential biocompatibility and are suitable for biointegration.

All BNC and RC meshes were easily identified at one- and three-month postimplantation. Although cellulose is considered biodegradable, it does not readily degrade *in vivo* due to the absence of cellulases in animals.^17^ By contrast, van Ho et al.^39^ observed degradation of cellulose nanocrystals after 12 weeks in a subcutaneous rat injection model. Based on our findings, we anticipate no or slow degradation of BNC and RC, making them suitable implants for applications in which prolonged structural integrity is desired, and degradation is not a requirement, such as alternatives to PP meshes in current use. Cellulose nanocrystals exhibit limited stability under moisture-rich conditions.^80^ They were not included in the present study, since their use would be restricted to reinforcing existing matrices rather than serving as the primary material.

The elasticity and strength of the RC films, CNF mesh, and BNC are all key factors in their suitability for different biomedical applications. The uniform texture of RC films and their mechanical properties make them suitable for applications where flexibility and smooth surface interaction are necessary. Meanwhile, the CNF mesh’s mechanical robustness and porosity make it more suitable to highly interactive tissue environments.^81^

## Conclusions

The biocompatibility of bacterial nanocellulose (BNC) and cellulose nanofibrils (CNF) was confirmed in this study. The biocompatibility of alkali-dissolved (NaOH/H2O, ZnO) regenerated cellulose (RC) was demonstrated *in vivo*. All implanted cellulose-derived materials exhibited expected, consistent host tissue responses to a foreign material, comparable to PP. However, the *in vivo* response differed: RC, with analogous microstructure to that of PP, showed similar, mild-to-moderate foreign body reaction (FBR), consisting mostly of macrophages and foreign body giant cells at one-month postimplantation, and a milder FBR at three months. On the contrary, the FBR detected with BNC implants was significantly more severe one-month postimplantation and with CNF at three months when compared to PP. The rough surface of CNF might have contributed to the pronounced late FBR. All implanted cellulose-derived meshes induced cell ingrowth. Differences in porosity allowed cells to penetrate through the pores of BNC, RC, and CNF, unlike the solid microstructure of PP and PDO. CNF showed extensive implant fragmentation and phagocytosis likely due to morphology effects and implantation in dry conditions.

All cellulose-derived materials showed potential for future clinical applications as scaffolds for tissue repair; however, the *in vivo* fragmentation observed for the CNF material needs to be taken into consideration if produced and used as in the current study. Our results provide valuable insights into mesh materials, laying a foundation for future *in vivo* investigations that should consider the specific morphologies, procedures and number of implants tested.

## Data Availability

The data of histopathological evaluation used to support the findings of this study is included within the Supporting Information file (S2).

## Supporting Information Available

Wound grading after subcutaneous implantation of bacterial nanocellulose (BNC), regenerated cellulose (RC), 3D-printed cellulose nanofibrils (CNFs), polypropylene (PP) and polydioxanone (PDO) in a rat subcutaneous transplantation model (PDF)
Cell response to different implants in a rat subcutaneous transplantation model (PDF)

## Abbreviations

BNC: bacterial nanocellulose
RC: regenerated cellulose
CNFs: cellulose nanofibrils
SEM: scanning electron microscopy
FBR: foreign body reaction
FBGC: foreign body giant cell
