# Supplementary material for: Comparative In Vivo Biocompatibility of Cellulose-Derived and Synthetic Meshes in Subcutaneous Transplantation Models
Source: Biomacromolecules. 2024 Oct 8;25(11):7298–310. doi: 10.1021/acs.biomac.4c00984 (PMC11558565; doi:10.1021/acs.biomac.4c00984)

Supporting Information S1:

Comparative *in vivo* biocompatibility of cellulose-derived and synthetic meshes in subcutaneous transplantation models

Nina M.M. Peltokallio[1,], Rubina Ajdary[2], Guillermo Reyes[2,*], Esko Kankuri[3], Jouni J.T. Junnila[4], Satu Kuure[5], Anna S. Meller[6], Jani Kuula[7], Eija Raussi-Lehto[7,8], Hannu Sariola[9], Outi M. Laitinen-Vapaavuori[1], Orlando J. Rojas[2,10,11,12,*]*

[1] Department of Equine and Small Animal Medicine, Faculty of Veterinary Medicine, University of Helsinki, Viikintie 49, FI-00014 Helsinki University, Finland.

[2] Biobased Colloids and Materials, Department of Bioproducts and Biosystems, School of Chemical Engineering, Aalto University, P.O. Box 16300, FI-00076 Aalto, Espoo, Finland.

[3] Department of Pharmacology, Faculty of Medicine, University of Helsinki, P.O. Box 29, Helsinki, Finland.

[4] EstiMates Oy, Kamreerintie 8, FI-022770 Espoo, Finland.

[5] GM unit, Helsinki Institute of Life Science/STEMM, Research Program´s Unit, Faculty of Medicine, University of Helsinki, P.O. Box 63, Helsinki, Finland.

[6] Laboratory Animal Centre, HiLIFE, University of Helsinki, P.O. Box 29, Helsinki, Finland

[7] Department of Neuroscience and Biomedical Engineering, School of Science, Aalto University, P.O. Box 16300, FI-00076 Aalto, Espoo, Finland.

[8] Customer-oriented Wellbeing and Health Services, Metropolia University of Applied Sciences, PL 4000, FI-00079 Metropolia, Helsinki, Finland.

[9] Department of Pathology, Faculty of Medicine, University of Helsinki, P.O. Box 63, Helsinki, Finland.

[10] Bioproducts Institute, Department of Chemical and Biological Engineering, The University of British Columbia, 2360 East Mall, Vancouver, BC V6T 1Z3, Canada.

[11] Department of Wood Science, University of British Columbia, 2385 East Mall, Vancouver, BC V6T 1Z4, Canada.

[12] Department of Chemistry, University of British Columbia, 2036 Main Mall, Vancouver, BC V6T 1Z1, Canada.

* **Corresponding authors:** Nina M.M. Peltokallio (Email: nina.peltokallio@helsinki.fi), Guillermo Reyes (guillermo.reyes@vtt.fi), Orlando J. Rojas (orlando.rojas@ubc.ca).

Figure S1. Wound grading after subcutaneous implantation of bacterial nanocellulose (BNC), regenerated cellulose (RC), 3D-printed cellulose nanofibrils (CNFs), polypropylene (PP) and polydioxanone in a rat subcutaneous transplantation model. Normal wound healing right after implantation of CNF and PP (a), sham and PP (b, c), RC and PP (d), and BNC and PP (e) at 0 (a), 1 (b), 7 (c), 30 (d), and 90 (e) days post-implantation. Cellulose-derived implants on the right, PP on the left side. Wound reactions classified as (f) mild swelling (PP), (g) clear discharge (RC, PP), and (h) abscess (PP).

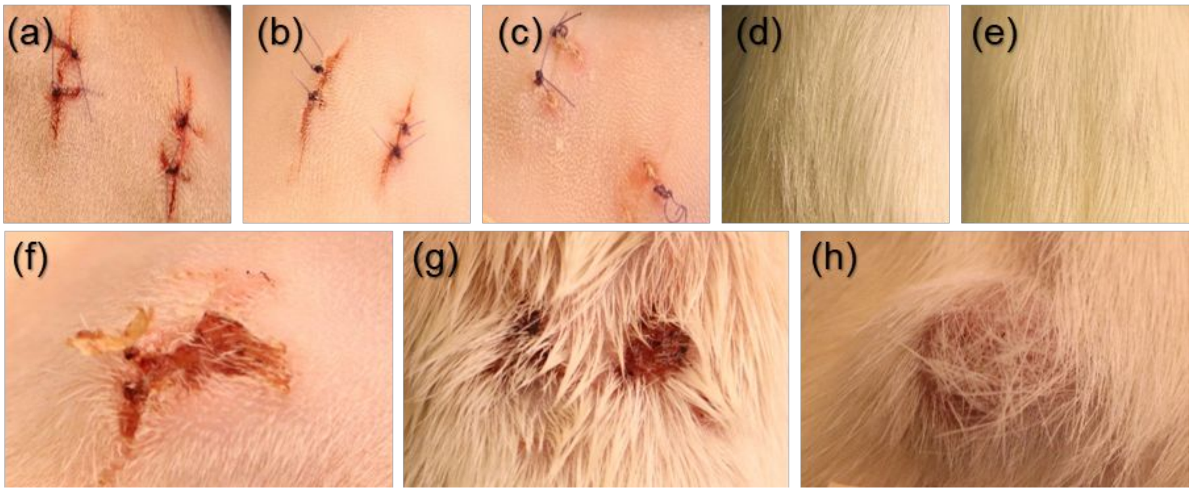

Supplement: Supplementary file 1 — bm4c00984_si_001.pdf [file bm4c00984_si_001.pdf]
