# Supplementary material for: Comparative In Vivo Biocompatibility of Cellulose-Derived and Synthetic Meshes in Subcutaneous Transplantation Models
Source: Biomacromolecules. 2024 Oct 8;25(11):7298–310. doi: 10.1021/acs.biomac.4c00984 (PMC11558565; doi:10.1021/acs.biomac.4c00984)
Supplement: Supplementary file 2 — bm4c00984_si_002.pdf [file bm4c00984_si_002.pdf]

Supporting Information S2:

Comparative *in vivo* biocompatibility of cellulose-derived and synthetic meshes in subcutaneous transplantation models

Nina M.M. Peltokallio[1,], Rubina Ajdary[2], Guillermo Reyes[2,*], Esko Kankuri[3], Jouni J.T. Junnila[4], Satu Kuure[5], Anna S. Meller[6], Jani Kuula[7], Eija Raussi-Lehto[7,8], Hannu Sariola[9], Outi M. Laitinen-Vapaavuori[1], Orlando J. Rojas[2,10,11,12,*]*

[1] Department of Equine and Small Animal Medicine, Faculty of Veterinary Medicine, University of Helsinki, Viikintie 49, FI-00014 Helsinki University, Finland.

[2] Biobased Colloids and Materials, Department of Bioproducts and Biosystems, School of Chemical Engineering, Aalto University, P.O. Box 16300, FI-00076 Aalto, Espoo, Finland.

[3] Department of Pharmacology, Faculty of Medicine, University of Helsinki, P.O. Box 29, Helsinki, Finland.

[4] EstiMates Oy, Kamreerintie 8, FI-022770 Espoo, Finland.

[5] GM unit, Helsinki Institute of Life Science/STEMM, Research Program´s Unit, Faculty of Medicine, University of Helsinki, P.O. Box 63, Helsinki, Finland.

[6] Laboratory Animal Centre, HiLIFE, University of Helsinki, P.O. Box 29, Helsinki, Finland

[7] Department of Neuroscience and Biomedical Engineering, School of Science, Aalto University, P.O. Box 16300, FI-00076 Aalto, Espoo, Finland.

[8] Customer-oriented Wellbeing and Health Services, Metropolia University of Applied Sciences, PL 4000, FI-00079 Metropolia, Helsinki, Finland.

[9] Department of Pathology, Faculty of Medicine, University of Helsinki, P.O. Box 63, Helsinki, Finland.

[10] Bioproducts Institute, Department of Chemical and Biological Engineering, The University of British Columbia, 2360 East Mall, Vancouver, BC V6T 1Z3, Canada.

[11] Department of Wood Science, University of British Columbia, 2385 East Mall, Vancouver, BC V6T 1Z4, Canada.

[12] Department of Chemistry, University of British Columbia, 2036 Main Mall, Vancouver, BC V6T 1Z1, Canada.

* **Corresponding authors:** Nina M.M. Peltokallio (Email: nina.peltokallio@helsinki.fi), Guillermo Reyes (guillermo.reyes@vtt.fi), Orlando J. Rojas (orlando.rojas@ubc.ca).

Figure S2: Cell response to different implants in a rat subcutaneous transplantation model

Follow-up	Rat no	Implanted material	FBR 1	FBR 2	FBR 3	FBR 4	AI	CI	GT	Scar	Excluded
**1 month**	3	RC	0	0	0	0	0	0	1	0	0
	3	PP	1	0	0	0	0	0	0	1	0
	4	CNF	1	0	0	0	0	0	0	0	0
	4	PP	1	0	0	0	0	0	0	0	0
	7	SHAM	1	0	0	0	0	0	0	0	0
	7	PP	0	0	0	0	0	0	0	1	0
	8	BC	1	0	0	0	0	0	0	0	0
	8	PP	0	0	0	0	0	0	1	0	0
	11	CNF	1	0	0	0	0	0	0	0	0
	11	PP	1	0	0	0	0	0	0	0	0
	12	SHAM	0	0	0	0	0	0	1	0	0
	12	PP	0	1	0	0	0	0	0	0	0
	15	BC	0	1	0	0	0	0	0	0	0
	15	PP	0	0	0	0	0	0	0	1	0
	16	RC	1	0	0	0	0	0	0	0	0
	16	PP	1	0	0	0	0	0	0	0	0
	19	SHAM	0	0	0	0	1	0	1	0	0
	19	PP	1	0	0	0	0	0	0	0	0
	20	BC	1	0	0	0	0	0	0	0	0
	20	PP	1	0	0	0	1	0	0	0	0
	23	RC	0	1	0	0	0	0	0	0	0
	23	PP	0	1	0	0	0	0	0	0	0
	24	CNF	0	0	1	0	0	0	0	0	0
	24	PP	0	0	0	0	0	0	0	1	0
	27	BC	1	0	0	0	0	0	0	0	0
	27	PP	0	0	0	0	0	0	0	1	0
	28	RC	1	0	0	0	0	0	0	0	0
	28	PP	0	1	0	0	0	0	0	0	0
	31	CNF	0	0	0	0	0	0	1	0	0
	31	PP	1	0	0	0	1	0	0	0	0
	32	SHAM	1	0	0	0	0	0	0	0	0
	32	PP	1	0	0	0	0	0	0	0	0
	35	RC	0	0	0	0	0	0	0	1	0
	35	PP	1	0	0	0	0	0	0	0	0
	36	CNF	0	1	0	0	0	0	0	0	0
	36	PP	1	0	0	0	0	0	0	0	0
	39	SHAM	0	1	0	0	0	0	0	0	0
	39	PP	1	0	0	0	0	0	0	0	0
	40	BC	0	0	1	0	0	0	0	0	0
	40	PP	1	0	0	0	0	0	0	0	0
	43	CNF	0	1	0	0	0	0	0	0	0
	43	PP	1	0	0	0	0	0	0	0	0
	44	SHAM	1	0	0	0	0	0	0	1	0

44	PP	0	0	0	0	0	0	0	0	1
47	BC	0	1	0	0	0	0	0	0	0
47	PP	0	0	0	0	1	0	0	0	0
48	RC	0	0	0	0	0	0	0	0	1
48	PP	1	0	0	0	0	0	0	0	0
51	SHAM	0	1	0	0	0	0	0	0	0
51	PP	1	0	0	0	0	0	0	0	0
52	BC	0	0	0	0	0	0	1	0	0
52	PP	0	0	0	0	0	1	1	0	0
55	RC	0	0	0	0	0	0	0	0	1
55	PP	1	0	0	0	0	0	0	0	0
56	CNF	0	0	0	0	0	0	0	0	1
56	PP	1	0	0	0	1	0	0	0	0
59	BC	0	1	0	0	0	0	0	0	0
59	PP	0	0	0	0	0	0	0	1	0
60	RC	1	0	0	0	1	0	0	0	0
60	PP	1	0	0	0	0	0	0	0	0
63	CNF	0	0	0	0	0	0	0	0	1
63	PP	0	0	0	0	0	0	1	0	0
64	SHAM	0	0	0	0	0	0	0	0	1
64	PP	1	0	0	0	0	0	0	0	0
67	RC	0	0	0	0	0	0	0	0	1
67	PP	1	0	0	0	0	0	1	0	0
68	CNF	0	0	0	0	1	0	0	0	0
68	PP	0	0	0	0	0	0	1	0	0
71	SHAM	0	0	0	0	0	0	0	0	1
71	PP	1	0	0	0	0	0	0	0	0
72	BC	0	0	0	0	0	0	0	0	1
72	PP	0	0	0	0	0	0	1	0	0
75	CNF	0	0	0	0	0	0	0	0	1
75	PP	0	1	0	0	0	0	0	0	0
76	SHAM	0	0	0	0	0	0	0	0	1
76	PP	0	0	0	0	0	0	1	0	0
79	BC	0	1	0	0	0	0	0	0	0
79	PP	0	1	0	0	0	0	0	0	0
80	RC	0	0	0	0	0	0	0	0	1
80	PP	0	1	0	0	0	0	0	0	0
		**32**	**15**	**2**	**0**	**7**	**1**	**12**	**8**	**12**

Follow-up	Rat no	Implanted material	FBR 1	FBR 2	FBR 3	FBR 4	AI	CI	GT	Scar	Missing
3 months	1	BC	0	0	0	0	0	0	0	0	1
	1	PP	0	0	0	0	0	0	0	0	1
	2	RC	0	0	0	0	0	0	0	0	1
	2	PP	0	0	0	0	0	0	0	0	1
	5	CNF	0	0	0	0	0	0	0	0	1
	5	PP	0	0	0	0	0	0	0	0	1
	6	SHAM	1	0	0	0	0	0	0	0	0
	6	PP	0	1	0	0	0	0	0	0	0
	9	RC	0	0	0	0	0	0	0	0	1
	9	PP	0	0	0	0	0	0	0	0	1
	10	CNF	0	0	0	0	0	0	0	0	1
	10	PP	0	0	0	0	0	0	0	0	1
	13	SHAM	0	1	0	0	0	0	0	0	0
	13	PP	0	0	0	0	0	0	1	0	0
	14	BC	0	0	0	0	0	0	0	0	1
	14	PP	0	0	0	0	0	0	0	0	1
	17	CNF	0	0	0	0	0	0	0	0	1
	17	PP	0	0	0	0	0	0	0	0	1
	18	SHAM	0	1	0	0	0	0	0	0	0
	18	PP	1	0	0	0	0	0	0	0	0
	21	BC	0	0	0	0	0	0	0	0	1
	21	PP	0	0	0	0	0	0	0	0	1
	22	RC	0	0	0	0	0	0	0	0	1
	22	PP	0	0	0	0	0	0	0	0	1
	25	SHAM	1	0	0	0	0	0	0	0	0
	25	PP	1	0	0	0	0	0	0	0	0
	26	BC	0	0	1	0	0	0	0	0	0
	26	PP	0	1	0	0	0	0	0	0	0
	29	RC	1	0	0	0	0	0	0	0	0
	29	PP	1	0	0	0	0	0	0	0	0
	30	CNF	1	0	0	0	0	0	0	0	0
	30	PP	0	1	0	0	0	0	0	0	0
	33	BC	1	0	0	0	0	0	0	0	0
	33	PP	0	1	0	0	0	0	0	0	0
	34	RC	1	0	0	0	0	0	0	0	0
	34	PP	0	0	0	0	0	0	0	1	0
	37	CNF	0	1	0	0	0	0	0	0	0
	37	PP	1	0	0	0	0	0	0	0	0
	38	SHAM	1	0	0	0	0	0	0	0	0
	38	PP	0	1	0	0	0	0	0	0	0
	41	RC	1	0	0	0	0	0	0	0	0
	41	PP	0	1	0	0	0	0	0	0	0
	42	CNF	0	0	1	0	0	0	0	0	0
	42	PP	0	1	0	0	0	0	0	0	0
	45	SHAM	0	0	0	0	0	0	0	0	1

45	PP	0	0	0	0	0	0	1	0	0
46	BC	1	0	0	0	0	0	0	0	0
46	PP	1	0	0	0	0	0	0	0	0
49	CNF	0	0	0	1	0	0	0	0	0
49	PP	0	0	1	0	0	0	0	0	0
50	SHAM	0	0	0	0	0	0	0	1	0
50	PP	0	1	0	0	0	0	0	0	0
53	BC	1	0	0	0	0	0	0	0	0
53	PP	0	1	0	0	0	0	0	0	0
54	RC	1	0	0	0	0	0	0	0	0
54	PP	1	0	0	0	0	0	0	0	0
57	SHAM	1	0	0	0	0	0	0	0	0
57	PP	0	0	0	0	0	0	1	0	0
58	BC	0	1	0	0	0	0	0	0	0
58	PP	1	0	0	0	0	1	0	0	0
61	RC	1	0	0	0	0	0	0	0	0
61	PP	0	0	0	0	0	0	0	1	0
62	CNF	0	0	0	0	0	0	0	0	1
62	PP	0	0	0	0	0	0	0	0	1
65	BC	1	0	0	0	0	0	0	0	0
65	PP	1	0	0	0	0	0	0	0	0
66	RC	1	0	0	0	0	0	0	0	0
66	PP	1	0	0	0	0	0	0	0	0
69	CNF	0	1	0	0	0	0	0	0	0
69	PP	0	1	0	0	0	0	0	0	0
70	SHAM	1	0	0	0	0	0	0	0	0
70	PP	1	0	0	0	0	0	0	0	0
73	RC	1	0	0	0	0	0	0	0	0
73	PP	0	0	0	0	0	0	0	1	0
74	CNF	0	0	1	0	0	0	0	0	0
74	PP	0	1	0	0	0	0	0	0	0
77	SHAM	0	0	0	0	0	0	0	0	1
77	PP	1	0	0	0	0	0	0	0	0
78	BC	0	1	0	0	0	0	0	0	0
78	PP	0	1	0	0	0	0	0	0	0
		**28**	**18**	**4**	**1**	**0**	**1**	**3**	**4**	**22**

FBR = foreign body reaction
AI = acute inflammation
CI = chronic inflammation
GT = granulation tissue
RC = regenerated cellulose
BC = bacterial nanocellulose

CNF = 3-D-printed cellulose nanofibrils
SHAM = polydioxanone (PDS®, Johnson&Johnson, USA)
PP = polypropylene
Excluded = non-representative samples, technical processing issues, premature euthanasia (1) due to study-unrelated cause
